# FluChip‐8G Insight: HA and NA subtyping of potentially pandemic influenza A viruses in a single assay

**DOI:** 10.1111/irv.12683

**Published:** 2019-10-13

**Authors:** Evan Toth, Erica D. Dawson, Amber W. Taylor, Robert S. Stoughton, Rebecca H. Blair, James E. Johnson, Amelia Slinskey, Ryan Fessler, Catherine B. Smith, Sarah Talbot, Kathy Rowlen

**Affiliations:** ^1^ InDevR, Inc. Boulder CO USA; ^2^ Influenza Division the Centers for Disease Control and Prevention Atlanta GA USA; ^3^ Present address: Arthrex California Technology Santa Barbara CA USA; ^4^ Present address: Kapteyn‐Murnane Laboratories Boulder CO USA; ^5^ Present address: Illumina, Inc. San Diego CA USA

**Keywords:** influenza, multiplex PCR, neural networks, oligonucleotide microarrays, pandemics, validation studies

## Abstract

**Background:**

Global influenza surveillance in humans and animals is a critical component of pandemic preparedness. The FluChip‐8G Insight assay was developed to subtype both seasonal and potentially pandemic influenza viruses in a single assay with a same day result. FluChip‐8G Insight uses whole gene segment RT‐PCR‐based amplification to provide robustness against genetic drift and subsequent microarray detection with artificial neural network‐based data interpretation.

**Objectives:**

The objective of this study was to verify and validate the performance of the FluChip‐8G Insight assay for the detection and positive identification of human and animal origin non‐seasonal influenza A specimens.

**Methods:**

We evaluated the ability of the FluChip‐8G Insight technology to type and HA and NA subtype a sample set consisting of 297 results from 180 unique non‐seasonal influenza A strains (49 unique subtypes).

**Results:**

FluChip‐8G Insight demonstrated a positive percent agreement ≥93% for 5 targeted HA and 5 targeted NA subtypes except for H9 (88%), and negative percent agreement exceeding 95% for all targeted subtypes.

**Conclusions:**

The FluChip‐8G Insight neural network‐based algorithm used for virus identification performed well over a data set of 297 naïve sample results, and can be easily updated to improve performance on emerging strains without changing the underlying assay chemistry.

## 1 INTRODUCTION

The annual estimates of morbidity and mortality caused by influenza A and B infections are 48.8 million illnesses, 959 000 hospitalizations, and 79 400 deaths in the United States in the 2017‐2018 season.^1^ Robust continual surveillance of circulating strains of influenza A and B which are subject to high rates of mutation is necessary to guide annual vaccine strain selection.^2^ In addition, the continued threat of pandemic influenza highlights the importance of continued and improved surveillance in both human and animal populations to better guide pandemic vaccine production and mitigate the potential impact of the global public health threat of pandemic influenza.^3^, ^4^, ^5^, ^6^, ^7^

Routine surveillance of influenza viruses often involves molecular assays based on real‐time RT‐PCR typically performed by clinical and public health laboratories. Though PCR‐based assays are sensitive, they typically rely on amplifying short portions of HA and NA, both of which exhibit high rates of genetic drift,^8^ with real‐time RT‐PCR assays having shown to be susceptible to reduced performance as genetic drift occurs.^9^, ^10^, ^11^, ^12^ Many real‐time RT‐PCR assays require numerous singleplex assays run serially, increasing the complexity and time to result for HA and NA subtyping. Microarray‐based approaches to influenza detection are also available. In addition to commercially available microarray‐based assays such as the ePLEX and eSensor RVP assays (both planar microarray assays, GenMark Diagnostics, Inc), the Verigene RV+ (planar microarray, Luminex Corp.), and the xTAG RVP assay (solution phase bead‐based microarray, Luminex Corp.), a variety of other microarray‐based approaches to influenza detection for both human and avian‐origin have appeared in the literature 13, 14, 15, 16, 17, 18, 19, 20, 21 but are not commercially available. Alternatively, next‐generation sequencing (NGS) of influenza viruses is routine in the three National Influenza Surveillance Reference Centers (NIRC) and at the Centers for Disease Control and Prevention (CDC), but has not been adopted for surveillance and other clinical microbiology applications in clinical and public health laboratories in part due to availability, cost, and complexity of data analysis.^2^, ^22^, ^23^

In this work, we report on a new assay called FluChip‐8G Insight for the detection and characterization of influenza viruses, including HA and NA subtyping for key potentially pandemic subtypes. FluChip‐8G Insight was developed with the goal of aiding public health, government, and academic laboratories conducting surveillance for pandemic influenza preparedness. The ability to differentiate influenza A viruses with high‐risk pandemic potential from currently circulating strains of influenza A is an important factor for influenza response preparedness and could enable more efficient detection of influenza viruses warranting immediate follow‐up analysis. The technology utilizes multiplexed RT‐PCR in which multiple full gene segments are amplified, followed by hybridization to a DNA microarray containing capture sequences that represent a significant fraction of the influenza A genome, and ultimate application of a modular, easily updateable neural network‐based pattern recognition to the microarray data. The benefits of this approach are universal amplification and detection of all influenza A and B viruses that provide robustness against genetic drift and shift, completely automated data interpretation, and the ability to rapidly update the underlying analysis algorithm to identify newly emerging viruses and to optimize performance as additional strains are obtained for inclusion in algorithm training.

FluChip‐8G Insight provides typing and subtyping of seasonal influenza A viruses and HA (H1, H3, H5, H7, and H9) and NA (N1, N2, N7, N8, and N9) subtyping of non‐seasonal A viruses in a single assay. Herein, we describe the verification of the HA and NA subtyping algorithm and subsequently assess performance in a validation study of 297 non‐seasonal influenza A samples.

## 2 MATERIALS AND METHODS

### 2.1 Viruses and characterization

All samples utilized in neural network algorithm optimization and subsequent testing were either archived original specimen material, grown viral isolates, or extracted nucleic acid (for highly pathogenic viruses or when no other material was available) of known type, subtype, and strain via either sequencing, real‐time RT‐PCR analysis, or via certificate of analysis obtained from a commercial vendor. All respiratory specimens obtained and utilized in this testing were completely de‐identified and provided without any protected health information. For several human clinical specimens, only subtype information was available. Contrived samples were prepared by spiking stock material into individual or pooled influenza‐negative human nasopharyngeal swab material stabilized in universal transport medium (UTM), and subsequently characterized by the CDC real‐time RT‐PCR influenza A typing assay to estimate concentration prior to analysis. All samples were stored at −70°C or below prior to executing the FluChip‐8G Insight assay.

### 2.2 FluChip‐8G Insight assay procedure

The FluChip‐8G Insight assay procedure was executed on all samples described herein according to the instructions for use. In brief, the Qiagen QIAamp MinElute Virus Spin Kit was utilized for nucleic acid extraction (200 µL starting volume, 50 µL elution volume). Extracted nucleic acid was then amplified using the QScript XLT One‐Step RT‐PCR kit (Quanta Biosciences). A dNTP mixture including biotin‐16‐aminoallyl‐2′ dUTP was used during RT‐PCR to incorporate biotin for downstream labeling of amplified products by a streptavidin‐coupled fluorophore. The RT‐PCR primers (provided as FC8G Primer Mix) provide multiplexed amplification of full influenza A gene segments HA, NA, M, NS, and NP, as well as full‐length influenza B HA and NA gene segments. In addition, for samples containing human genetic material a segment of the 18S rRNA found in eukaryotic cells was amplified as an internal control to assess sample integrity. RT‐PCR products were subsequently heat fragmented and hybridized to the microarray containing 458 influenza‐targeted short oligonucleotide capture sequences designed to target subtype‐ and lineage‐specific sequences of the 7 amplified gene segments. The microarray was subsequently washed, labeled, and imaged using the fluorescence‐based FluChip‐8G Imaging System (InDevR, Inc).

### 2.3 Neural network training

**Figure 1:**
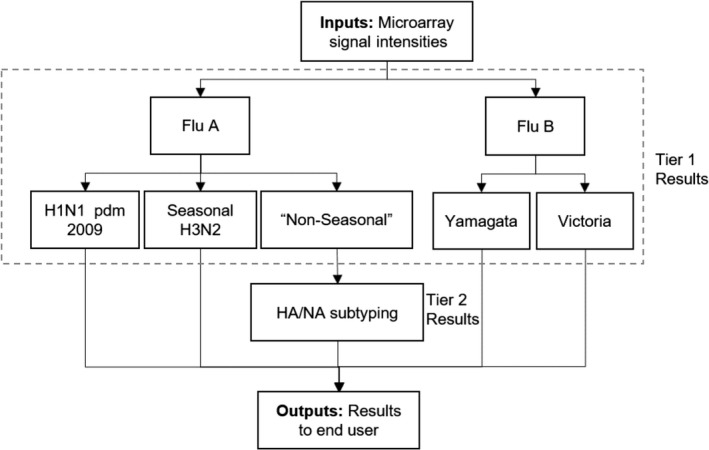
High‐level neural network algorithm architecture

Two^2^ tiers of artificial neural networks were used for pattern recognition‐based identification (see Supplementary Material), as schematically illustrated in Figure 1. All neural networks were trained to identify FluChip‐8G microarray patterns resulting from the 458 capture sequences using Fast Artificial Neural Network (FANN) open‐source library version 2.20 (http://leenissen.dk/fann/wp/). Tier 1 networks were trained to identify influenza A, H1N1 pandemic 2009, and seasonal H3N2, and to differentiate these from non‐seasonal influenza A. Tier 1 networks were also trained to identify influenza B Victoria and Yamagata lineages. This was accomplished with 3005 microarray images (samples) representing 415 unique strains of influenza (see Supplementary Tables S1 and S2). Tier 2 neural networks were trained to identify “non‐seasonal A” subtypes H1, H3, H5, H7, H9, N1, N2, N7, N8, and N9 as well as general “Hx” and “Nx” categories that included all other subtypes not specifically listed. Training of tier 2 networks was performed using 1479 microarray images (samples) representing 140 unique influenza A strains (see Supplementary Tables S3 and S4).

The neural networks were trained in 3 steps: First, the samples were split into 10 groups of approximately equal size and composition. Second, 10‐fold cross‐validation was completed by training neural networks utilizing sample groups 1‐9 with group 10 reserved for testing the newly trained networks. This process was repeated 9 times with a different group held out for testing each time. The results of the 10‐fold cross‐validation were then examined to assess the ability of each of the groups of trained neural networks to predict the expected sample result (see Results section). Lastly, final training was completed using all ten^10^ groups to produce the optimized neural network algorithm. Upon successful training, tier 1 and tier 2 network parameters were coded into the FluChip‐8G Insight Software which was subsequently loaded onto the FluChip‐8G Imaging System for validation testing using a completely naïve sample test set.

### 2.4 Naïve test set composition

The test set used for evaluation of Tier 2 network performance was made up of 297 microarray images each representing a single contrived sample processed by the FluChip‐8G Insight assay procedure. These samples encompassed 180 unique strains representing 49 unique subtypes (Supplementary Table S3) and were comprised of human samples and grown isolates or extracted nucleic acid of avian, porcine, and equine strains from North America, South America, Europe, and Asia. All 180 unique strains included were samples known to be “non‐seasonal” influenza A via the methods described above. Of the 297 total images, 280 were identified as “influenza A, non‐seasonal” by the tier 1 networks and therefore were analyzed by the tier 2 networks for subtype identification. Performance data for tier 2 networks therefore represent only sample images correctly identified as “non‐seasonal” influenza A by the tier 1 networks.

## 3 RESULTS

### 3.1 Algorithm optimization via 10‐fold cross‐validation

All of the virus samples included in the tier 2 neural network training set (see Supplementary Table S3) were processed by the FluChip‐8G Insight assay, with the microarray images subsequently utilized to perform 10‐fold cross‐validation of the tier 2 neural network algorithm. Performance of the 10‐fold cross‐validation of the training set is shown in Table 1 below. As described previously, this training set was comprised of only samples known to be positive for non‐seasonal influenza A.

**Table 1:** Results of 10‐fold cross‐validation of the tier 2 neural network training set

Subtype	Total samples	Total unique strains	Positive agreement		Negative agreement	
			TP/(TP + FN)	% ± 95% CI	TN/(TN + FP)	% ± 95% CI
H1	353	24	346/(346 + 7)	98% ± 1%	1119/(1119 + 7)	99% ± 0%
H3	290	46	281/(281 + 9)	97% ± 2%	1183/(1183 + 6)	100% ± 0%
H5	279	24	276/(276 + 3)	99% ± 1%	1193/(1193 + 7)	99% ± 0%
H7	237	13	230/(230 + 7)	97% ± 2%	1238/(1238 + 4)	100% ± 0%
H9	209	11	205/(205 + 4)	98% ± 2%	1266/(1266 + 4)	100% ± 0%
Hx (all other)	111	26	103/(103 + 8)	93% ± 5%	1364/(1364 + 4)	100% ± 0%
N1	447	35	432/(432 + 15)	97% ± 2%	1016/(1016 + 16)	98% ± 1%
N2	471	46	455/(455 + 16)	97% ± 2%	993/(993 + 15)	99% ± 1%
N7	115	10	113/(113 + 2)	98% ± 2%	1363/(1363 + 1)	100% ± 0%
N8	212	24	203/(203 + 9)	96% ± 3%	1265/(1265 + 2)	100% ± 0%
N9	169	12	162/(162 + 7)	96% ± 3%	1307/(1307 + 3)	100% ± 0%
Nx (all other)	65	63	61/(61 + 4)	94% ± 6%	1410/(1410 + 4)	100% ± 0%

All HA and NA neural networks designed to detect specific subtypes demonstrated >95% positive percent agreement (a measure of the frequency of false negatives) and >98% negative percent agreement (a measure of the frequency of false positives). The “Hx” and “Nx” neural networks which are trained collectively on all subtypes other than those specifically addressed by another network (eg, H2, H4, and H6) demonstrated lower agreement overall, but both were still >92% positive agreement.

### 3.2 Assay performance assessment on naïve sample test set

To independently validate performance of the FluChip‐8G Insight assay and algorithm, the naïve sample test set of contrived samples (see Supplementary Table S5) was processed using the FluChip‐8G Insight assay and the data subsequently analyzed using the optimized neural network algorithm. The result of each specimen processed was compared to the expected result to assess the positive and negative percent agreement shown in Table 2.

**Table 2:** Performance data on 280 samples from the tier 2 neural network test set

Subtype	Total samples	Total unique strains	Positive agreement		Negative agreement	
			TP/(TP + FN)	% ± 95% CI	TN/(TN + FP)	% ± 95% CI
H1	26	15	26/(26 + 0)	100% ± 0%	253/(253 + 1)	100% ± 1%
H3	37	22	36/(36 + 1)	97% ± 5%	238/(238 + 5)	98% ± 2%
H5	69	57	67/(67 + 2)	97% ± 4%	211/(211 + 0)	100% ± 0%
H7	39	28	38/(38 + 1)	97% ± 5%	232/(232 + 9)	96% ± 2%
H9	33	17	29/(29 + 4)	88% ± 11%	247/(247 + 0)	100% ± 0%
Hx (all other)	76	41	58/(58 + 18)	76% ± 10%	202/(202 + 2)	99% ± 1%
N1	86	60	86/(86 + 0)	100% ± 0%	194/(194 + 2)	99% ± 1%
N2	67	42	63/(63 + 4)	94% ± 6%	205/(205 + 10)	95% ± 3%
N7	19	11	18/(18 + 1)	95% ± 10%	263/(263 + 0)	100% ± 0%
N8	16	11	15/(15 + 1)	94% ± 12%	266/(266 + 0)	100% ± 0%
N9	20	12	20/(20 + 0)	100% ± 0%	261/(261 + 1)	100% ± 1%
Nx (all other)	74	44	59/(59 + 15)	80% ± 9%	207/(207 + 1)	100% ± 1%

For HA and NA subtyping, positive percent agreement for all classifications targeting specific subtypes exceeded 93%, except for H9 which resulted in 87.9% positive percent agreement. Similar to the results seen for the 10‐fold cross‐validation shown in Table 1, the “Hx” and “Nx” categories demonstrated lower positive percent agreement of 76.3% and 79.7%, respectively. Negative percent agreement for HA and NA subtypes targeted exceeded 95%.

## 4 DISCUSSION

High positive percent agreement over the training set via 10‐fold cross‐validation (Table 1) indicates both that the images used for training were appropriate and that the algorithm optimization was successful. The poorest performing categories were, unsurprisingly, the Hx and Nx classifications. This reduced performance is due to the wide subtype diversity in this classification, as well as the relatively low number of strains available for each of these less common subtypes. For example, the makeup of the Hx training set consists of 4 H2, 4 H4, 2 H6, 4 H10, 7 H11, 1 H12, 1 H13, and 1 H16 strains. That is, the single subtype HA and NA networks are on average trained with 25 unique strains of a specific HA or NA subtype, while each subtype in the Hx and Nx is on average trained with only 4 unique strains of their subtype due to availability.

While high concordance achieved during 10‐fold cross‐validation is a good indicator of the appropriateness of the algorithm to predict performance on a generalized data set, a true challenge of the system is the ability to achieve high concordance for a set of samples to which the neural network algorithm is naïve. Seventeen^17^ out of 297 samples (6%) from the tier 2 network test set were not identified as non‐seasonal influenza A, by the tier 1 neural networks, and therefore, only the 280 correctly identified sample images from the test set were evaluated by the tier 2 networks reported in Table 2. These 17 samples likely failed to be identified as non‐seasonal influenza A due to the low concentration of influenza present. Fourteen^14^ out of 17 of these samples resulted in a call of “influenza not detected” (influenza A typing neural network returned a negative result), indicating that these samples were likely below the FluChip‐8G Insight assay limit of detection (LOD) which corresponds to a CDC real‐time RT‐PCR influenza A cycle threshold (Ct) value of approximately 32. The remaining 3 samples were only identified as positive for influenza A, meaning the A typing neural network returned a positive result, but the subtyping networks returned a negative result. Because the subtyping neural networks are slightly less sensitive than the tier 1 neural networks, this behavior, typing with no subtyping, indicates that these samples are likely just below the LOD.

All of the specific HA and NA subtypes targeted resulted in negative percent agreement (NPA) ≥95.3%, indicating the assay is sufficiently robust against false‐positive results. In this instance, false positives are samples that get misidentified as another non‐seasonal HA or NA subtype (as all included in this analysis were positively identified as non‐seasonal A by the tier 1 networks). Positive percent agreement (PPA) was ≥93.8% for all targeted HA and NA subtypes except for H9, which resulted in PPA of 87.9%. The lower PPA for H9 samples was caused by the failure to correctly identify 4 H9N7 samples. It is likely that FluChip‐8G Insight is less sensitive to H9N7 because of a training database imbalance for H9 samples, leading to misinterpretation of the microarray pattern. Specifically, 94% of the H9 sample images included in training came from analysis of H9N2 samples, whereas only 4% came from the analysis of H9N7 samples. This indicates that the neural networks do not correctly identify the microarray pattern created by the H9 from an H9N7 specimen because they are biased to identify H9 from H9N2 lineages. H9 sample identification would likely improve if more diverse samples comprised of the H9 HA subtype were processed with the FluChip‐8G Insight assay and added to the training database to update the neural network parameters.

The Hx and Nx classifications exhibited the lowest performance in this naïve sample set. This behavior was expected given the similar performance observed for the 10‐fold cross‐validation shown in Table 1. Again, this reduced performance is likely due to the increased subtype diversity within this group that is trained on a variety of “other” less commonly encountered subtypes (for which samples to test and include in training are less available). For example, 12 of the 32 (38%) unique subtypes in the naïve sample set that fall into either the Hx or Nx category were not included in the tier 2 network training set. Performance would very likely be further improved for all classifications by processing and training additional images representing a range of strains and concentrations for each target subtype as new subtypes and strains become available.

The ability of the algorithm to be easily updated and re‐verified (and the software subsequently updated) as additional samples are analyzed highlights the power of a universal amplification scheme coupled with a neural network‐based data analysis approach. For example, if a certain subtype currently included only in the Hx category emerged as an important pathogen, such as H11, additional samples of this new subtype of interest could be processed by the FluChip‐8G Insight assay. The resulting data could then be used to re‐architect and update the algorithm, and new software could be released relatively quickly. Similarly, if a surveillance site detects numerous Hx or Nx specimens, the user could analyze the specimens via NGS, and again, the neural networks could be updated to specifically detect this subtype of interest. Importantly, these updates to assay performance could be executed *without any redesign of the underlying assay chemistry required*. Likewise, as additional samples are routinely analyzed, the resulting microarray data can be fed back into the training process to drive further improvement in the algorithm performance.

Given ongoing genetic drift of influenza viruses and limited availability of next‐generation sequencing technology in routine influenza surveillance, the FluChip‐8G Insight assay could be used to continually screen circulating strains for both typical seasonal and potentially pandemic subtypes. If a new influenza A virus emerged with potentially pandemic importance, the algorithm and software could be rapidly updated to offer improved detectability. This platform can therefore provide effective surveillance of currently circulating strains, while also rapidly identifying potentially pandemic viruses that could be quickly triaged for more extensive follow‐up such as next‐generation sequencing.

## CONFLICT OF INTEREST

ET, EDD, AWT, RSS, RHB, JEJ, AS, RF, and KR are current or former employees of InDevR. EDD and KR are stockholders of InDevR, Inc EDD, AWT, RSS, RHB, and KR are named inventors on patent applications related to the material herein.

## References

[1] cdc.gov [internet]. Centers for disease control and prevention; [cited 2019 Apr 3]. https://www.cdc.gov/flu/about/burden/2017-2018.htm

[2] Jester B , Schwerzmann J , Mustaquim D , et al. Mapping of the US Domestic influenza virologic surveillance landscape. Emerg Inf Dis. 2018;24(7):1300‐1306. 10.3201/eid2407.180028 PMC6038762 29715078

[3] Jester B , Uyeki T , Jernigan D . Readiness for responding to a severe pandemic 100 years after 1918. Amer. J. Epidemiol. 2018;187(12):2596‐2602. 30102376 10.1093/aje/kwy165 PMC7314205

[4] Webster RG . Are we ready for pandemic influenza? Science. 2003;302:1519‐1522. 14645836 10.1126/science.1090350

[5] Von Dobschuetz S , DeNardi M , Harris KA , et al. FLURISK consortium. Influenza surveillance in animals: what is our capacity to detect emerging influenza viruses with zoonotic potential? Epidemiol. Infect. 2015;143(10):2187‐2204. 25268692 10.1017/S0950268814002106 PMC9506977

[6] Uyeki TM , Katz JM , Jernigan DB . Novel influenza A Viruses and pandemic threats. Lancet. 2017;389(10085):2172‐2174. 28589883 10.1016/S0140-6736(17)31274-6 PMC6637738

[7] Perspective SK . Perspective: Ill prepared for a pandemic. Nature. 2014;507:S20‐S21. 24611175

[8] Steinhauer DA , Skehel JJ . Genetics of influenza viruses. Ann. Rev. Genet. 2002;36:305‐332. 12429695 10.1146/annurev.genet.36.052402.152757

[9] Stellrecht K . History of matrix gene mutations within PCR target regions among circulating influenza H3N2 clades over ten‐plus‐years. J Clin Virol. 2018;107:11‐18. 30103162 10.1016/j.jcv.2018.08.002

[10] Huzly D , Korn K , Bierbaum S , et al. Influenza A virus drift variants reduced the detection sensitivity of a commercial multiplex nucleic acid amplification assay in the season 2014/15. Arch Virol. 2016;161(9):2417‐2423. 27316440 10.1007/s00705-016-2930-8

[11] Yang J‐R , Kuo C‐Y , Huang H‐Y , et al. Newly emerging mutations in the matrix genes of the human influenza A(H1N1)pdm09 and A(H3N2) viruses reduce the detection sensitivity of real‐time reverse transcription‐PCR. J Clin Microbiol. 2014;52(1):76‐82. 24153120 10.1128/JCM.02467-13 PMC3911422

[12] Lee CK , Chiu LL , Loh TP , Koay ES . Missed detection of an avian influenza A (H7N9) virus by the Luminex xTAG RVP FAST version 2. Pathology. 2017;49(3):330‐332. 28279476 10.1016/j.pathol.2016.11.014

[13] Heydarov RN , Lomakina NF , Boravleva EY , et al. The use of microarrays for the identification of the origin of genes of avian influenza viruses in wild birds. Microbiol Indepen Res. J. 2017;4(1):21‐30.

[14] Shi L , Sun JS , Yang ZP , et al. Development of a DNA microarray‐based multiplex assay of avian influenza virus subtypes H5, H7, H9, N1, and N2. Acta Virolog. 2014;58:14‐19. 10.4149/av_2014_01_14 24717024

[15] Wang Y , Qu J , Ba Q , et al. Detection and typing of human‐infecting influenza viruses in china by using a multiplex DNA biochip assay. J Virol Meth. 2016;234:178‐185. 10.1016/j.jviromet.2016.04.021 27150046

[16] Nguyen V‐T , Nimse SB , Song K‐S , et al. HPAI 9G DNA chip: discrimination of highly pathogenic avian influenza viruses. Chem Comm. 2012;48:4582‐4584. 22456544 10.1039/c2cc30709j

[17] Li X , Qi X , Miao L , et al. Detection and subtyping of influenza A virus based on a short oligonucleotide microarray. Diag Microbiol Inf Dis. 2009;65(3):261‐270. 10.1016/j.diagmicrobio.2009.07.016 19733996

[18] Ryabinin VA , Kostina EV , Maksakova GA , Neverov AA , Chumakov KM , Sinyakov AN . Universal oligonucleotide microarray for sub‐typing of influenza A virus. PLoS ONE. 2011;6(4):1‐11. 10.1371/journal.pone.0017529 PMC3084687 21559081

[19] Mukherjee S , Chakarbarti AK . Impact of microarray technology in influenza virus research and diagnostics. J Proteomics Bioinform S. 2012;6:2.

[20] Paulin LF , de los D Soto‐Del Río M , Sánchez I et al. PhyloFlu, A DNA microarray for determining the phylogenetic origin of influenza A virus gene segments and the genomic fingerprint of viral strains. J Clin Microbiol. 2014;52(3):803‐813. 24353006 10.1128/JCM.03134-13 PMC3957772

[21] Gall A , Hoffmann B , Harder T , Grund C , Höper D , Beer M . Design and validation of a microarray for detection, hemagglutinin subtyping, and pathotyping of avian influenza viruses. J Clin Micriobiol. 2009;47(2):327‐334. 10.1128/JCM.01330-08 PMC2643657 19052173

[22] Balloux F , Brynildsrud OB , van Dorp L , et al. From theory to practice: translating whole‐genome sequencing (WGS) into the clinic. Trends Microbiol. 2018;26(12):1035‐1048. 30193960 10.1016/j.tim.2018.08.004 PMC6249990

[23] Hampson A , Barr I , Cox N , et al. Improving the selection and development of influenza virus vaccines–report of a WHO informal consultation on improving influenza vaccine virus selection, Hong Kong SAR, China, 18–20 November 2015. Vaccine. 2017;35:1104‐1109. 28131392 10.1016/j.vaccine.2017.01.018 PMC5357705

