# Supplementary material for: FluChip‐8G Insight: HA and NA subtyping of potentially pandemic influenza A viruses in a single assay
Source: Influenza Other Respir Viruses. 2019 Oct 13;14(1):55–60. doi: 10.1111/irv.12683 (PMC6928037; doi:10.1111/irv.12683)
Supplement: Supplementary file 1 [file IRV-14-55-s001.docx]

**Supplementary Material:** FluChip-8G Insight: HA and NA subtyping of potentially pandemic influenza A viruses in a single assay

**Neural Network Methodology/Architecture**

Artificial neural networks are algorithms for identifying complex interactions between inputs and outputs. They are utilized for tasks like classification and pattern recognition. A network learns how inputs are related to defined output categories through error minimization on a known ‘training set’ for which the optimal mathematical relationships connecting the inputs and the outputs are determined via back propagation. Once established, the optimized parameters can be used to predict the outputs for an unknown dataset. The algorithm training process can then be validated via 10-fold cross-validation. 10-fold cross-validation is a mathematical process commonly utilized employing known data to predict how well a machine learning algorithm will perform over an unknown dataset. The ‘training set’ is first divided into k sections; in this case, the data was parsed into ten subsets of approximately equal size and composition. Nine subsets were then used to train the neural network algorithm, with the tenth subset queried as unknowns (the “test” subset). Each combination of 9 training subsets and 1 test subset are utilized to train a network, so that each sample exists in the test subset for one train/test combination. The ability of the training set to predict the known outputs for the test subset are then assessed by calculating concordance with the expected results.

***Tier 1 Neural Networks.***

***Architecture.*** The first tier of 7 neural networks (shown schematically as ‘Tier 1’ in main article Figure 1) each answer a single question: does the FluChip-8G Insight data in question indicate positive or negative for each of 7 target virus groups? First, two networks independently identify whether or not a sample is positive for influenza A or influenza B. If a sample is identified as containing influenza A, the signal intensities are then independently evaluated by three additional neural networks for the presence of H1N1 pandemic 2009, seasonal H3N2, and ‘non-seasonal’ influenza A (defined as any influenza A not categorized as either human-origin H1N1 pandemic 2009 or human-origin H3N2). Alternatively, if a sample is identified to contain influenza B, signal intensities are evaluated for the presence of the Yamagata or Victoria lineage.

***Training***. Individual neural networks were optimized (trained) using Fast Artificial Neural Network (FANN) open source library version 2.20 (<http://leenissen.dk/fann/wp/>). A total of 3005 images representing 418 unique influenza strains (see Table S1) which in turn represented 63 unique influenza A subtypes (Table S2) were utilized in the training of the Tier 1 neural networks.

The entire training set was used for optimization of the Flu A, Flu B, and Non-Seasonal A networks, whereas the H1N1pdm 2009 and the seasonal H3N2 networks were optimized only with samples known to be Flu A positive. Likewise, the Yamagata and Victoria lineage networks were trained solely with samples known to be Flu B positive. All 458 influenza-targeted microarray capture sequence intensities were used as inputs, with a single hidden layer consisting of 1 hidden node, and a single output. The variables optimized during the training process were 458 weight factors applied to the inputs, the hidden node offset and gain, and the output node offset value. The hidden node numerical output feeds into a single output node that utilizes a sigmoid function to generate a smooth output value ranging from 0 to 1. Optimization of the algorithm was considered complete when each sample in the training set that should have been positive for a given net reached and output value greater than 0.997 and each sample in the training set that should be negative for that same net reached an output value lower than 0.003. After training was complete, the optimal threshold for each network was determined using receiver operator characteristics (ROC) curves and applied to the output of each network, with the output being either negative (below the threshold) or positive (above the threshold). The optimized algorithm post-training and threshold determination was subsequently coded in the custom FluChip-8G Insight software.

***Tier 2 Neural Networks***

***Architecture.*** For samples identified as a ‘non-seasonal’ influenza A by the tier 1 networks, data is then analyzed via a second tier of differently architected neural networks designed to perform HA and NA subtyping, as schematically shown in Figure 1 (see main article). In the second tier networks, a hybrid error-correcting redundancy scheme is used *(1).*  Briefly and using HA as an example, separate single neuron networks were trained to identify H1, H3, H5, H7, H9, and “Hx” (all other HA subtypes) independently. In addition, single neuron networks were trained to identify either subtype in a pair (i.e., H1 or H3, H1 or H5, H5 or H7 for every combination of two subtypes). The single and paired subtype networks yield a total of 21 independent HA-directed networks. An analogous set of single and paired subtype networks are utilized for NA to identify N1, N2, N7, N8, N9, and “Nx” (all other NA subtypes). If perfectly identified, any given sample should have an output of 1 for 6 of the 21 networks, and an output of 0 for the other 15. The expected output value of each network for a sample of a given subtype (classification) becomes the “codeword” for that subtype. The codewords for each subtype classification for HA are shown in Figure S1. For example, the HA3 network is trained to give a high output value (close to 1) for all samples of subtype H1 or H7. Similarly, any H7 sample should give an output close to 1 for the H7, HA3, HA7, HA10, HA13 & HA14 networks. Unlike the threshold used for classification in tier 1 networks, tier 2 networks classify a given sample based on its L1 distance from each subtype codeword, where the L1 distance is the sum over all 21 networks of the absolute values of the differences between the actual network outputs and codeword for that subtype. If the L1 distance for any classification (i.e. H1) is less than 5, then this sample is considered positive for that classification. Note that no 2 classifications can have an L1 distance less than 5 for any given sample.


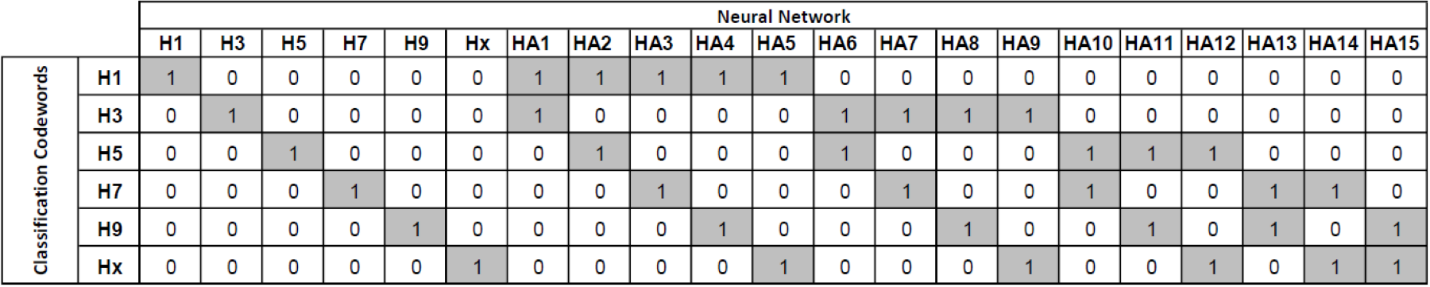


**Figure S1**: Neural network architecture showing ‘codeword’ for each non-seasonal subtype shown in the lefthand column. The codeword indicates which of the 21 neural networks shown should be positive (1) or negative (0) for each non-seasonal influenza A

***Training***. Tier 2 training of the (HA/NA subtyping) neural networks utilized 1479 microarray images representing 140 unique strains (see Table S3) representing 42 unique subtypes (see Table S4). The total number of images is larger than the number of unique strains because a strain was often utilized to prepare samples in different matrices or at different dilutions.

Since the tier 2 neural networks are intended to determine the HA and NA subtypes, the 21 redundant networks used to determine the HA and NA subtypes were optimized/trained on only signals from the 124 HA-targeted capture sequences and the 189 NA-targeted capture sequences, respectively. This was done for 2 reasons, first to prevent viruses containing reassorted M, NS or NP gene segments from confounding the identification of the HA and NA subtypes, and second, to prevent HA and/or NA bias based on training set composition. For example, the large number of H1N1 viruses could bias the N1 networks to classify and H1 sample as N1. (i.e. H1N2 incorrectly classified as H1N1). Other than this change to the inputs utilized in the Tier 2 networks, the training process utilized was the same as for the Tier 1 networks.

**References**

1. Dietterich T, Bakiri, G, Solving Multiclass Learning Problems via Error-Correcting Output Codes. J Artificial Intelligence Res. 1995; 2:263-286.

| **Table S1:** List of influenza A strains included in training tier 1 neural networks | | |
| --- | --- | --- |
| A/Alabama/19/2013 | A/Pennsylvania/16/2014 | A/Ohio/02/2012 |
| A/Brazil/9061/2014 | A/Pennsylvania/17/2014 | A/Oregon/04/2012 |
| A/California/01/2014 | A/Pennsylvania/18/2014 | A/Perth/16/2009 |
| A/California/04/2009 | A/Pennsylvania/19/2014 | A/Texas/06/2014 |
| A/California/07/2009 | A/Pennsylvania/20/2014 | A/Texas/12/2007 |
| A/CA/12/2012 H275 | A/Pennsylvania/21/2014 | A/Texas/24/2010 |
| A/California/59/2013 | A/Perth/261/2009 mut H275Y | A/Victoria/361/2011 |
| A/Colorado/07/2014 | A/Perth/265/2009 wt 275H | A/WA/01/2007 E119 |
| A/Delaware/04/2011 | A/Texas/100/2013 | A/Washington/33/2014 |
| A/Hawaii/06/2014 | A/Texas/23/2012 H275Y | A/Wisconsin/67/2005 |
| A/Indiana/1027/2013 | A/Uganda/MUWRP-176/2010 | A/Wyoming/09/2010 |
| A/Louisiana/10/2013 | A/Utah/02/2014 | A/Aichi/2/68 |
| A/Louisiana/13/2013 | A/Virginia/04/2014 | A/American Green-Winged Teal/Mississippi/300/2010 |
| A/Louisiana/35/2013 | A/Virginia/06/2014 | A/American Oystercatcher/Chile/C1307/2105 |
| A/Louisiana/36/2013 | A/Wisconsin/04/2014 | A/Anhui/1/2013 |
| A/Louisiana/37/2013 | A/Wisconsin/12/2013 | A/Anhui/2/2005 |
| A/Louisiana/41/2013 | A/Alabama/05/2012 | A/Aquatic Bird/Hong Kong/D125/2002 |
| A/Maryland/06/2011 | A/Alaska/03/2012 | A/Arizona/07/2007 |
| A/Mexico/818/2014 | A/Brisbane/10/2007 | A/Australian Shelduck/Western Australia/1756/83 |
| A/Mississippi/29/2013 | A/California/16/2011 | A/Avian/New York/11678-4/2005 |
| A/Mississippi/30/2013 | A/Florida/22/2009 | A/Black Headed Gull/Hong Kong/84/2012 |
| A/Missouri/02/2014 | A/Fukui/20/2004 wt 119E | A/Black-Legged Kittiwake/Quebec/02838-1/2009 |
| A/Netherlands/2290/2009 | A/Fukui/45/2004 mut E119V | A/Blue-Winged Teal/Alberta/346/2007 |
| A/New York/01/2009 | A/Kenya/6063/2010 | A/Blue-Winged Teal/Illinois/10Os1546/2010 |
| A/New York/33/2013 | A/Massachusetts/06/2010 | A/Blue-Winged Teal/Illinois/10Os1563/2010 |
| A/North Carolina/04/2014 | A/Nevada/09/2012 | A/Blue-Winged Teal/Iowa/10Os2411/2010 |
| A/North Carolina/21/2013 | A/Nevada/10/2012 | A/Blue-Winged Teal/Texas/G77/2007 |
| A/North Carolina/59/2009 | A/New Hampshire/05/2012 | A/Brisbane/59/2007 |
| A/Paraguay/901/2010 | A/New Jersey/01/2011 | A/Duck/Hokkaido/69/2000 |
| A/Pennsylvania/03/2014 | A/New Mexico/07/2012 | A/Duck/Hong Kong/Y280/1997 |
| A/Pennsylvania/14/2014 | A/North Carolina/05/2011 | A/Duck/Kulon Progo/Bb Vet/Ix/2004 |
| A/Camel/Mongolia/335/2012 | A/Chicken/Hong Kong/G9/1997 | A/Duck/Memphis/546/1974 |
| A/Canada/Rv444/2004 | A/Chicken/Hong Kong/Nt10/2011 | A/Duck/Minnesota/1525/1981 |
| A/Canine/Florida/89911,2/2006 | A/Chicken/Hong Kong/Yu341/2008 | A/Duck/Nanchang/1944/1993 |
| A/Canine/NY/100525,1/2006 | A/Chicken/Hunan/2246/2006 | A/Duck/Potsdam/S28716/1988 |
| A/Canine/New York/4732/2012 | A/Chicken/Italy/1285/2000 | A/Duck/Ukraine/1963 |
| A/Canine/Virginia/93653/2009 | A/Chicken/Italy/312/1997 | A/Dunlin/Delaware/281/2015 |
| A/Chelyabinsk/01/2006 | A/Chicken/Italy/9097/1997 | A/Egret/Egypt/1162-Namru3/2006 |
| A/Chick/Germany/N/49 | A/Chicken/Karachi/Narc-100/2004 | A/Environment/Bangladesh/10306/11 |
| A/Chicken/Bangladesh/10450/2011 | A/Chicken/New York/116124/2003 | A/Environment/Vietnam/Ncvdcdc53/2005 |
| A/Chicken/Bangladesh/18247/2012 | A/Chicken/Qalubia-Egypt/1/2006 | A/Environment/Vietnam/Ncvdcdc54/2005 |
| A/Chicken/Bangladesh/23974/2014 | A/Chicken/Saskatchewan/Hr10/2007 | A/Equine/Kentucky/4/2011 |
| A/Chicken/Bangladesh/25947/2015 | A/Chicken/Vietnam/Ncvdcdc33/2005 | A/Equine/New York/452/2003 |
| A/Chicken/Beijing/1/1994 | A/Chicken/Vietnam/Ncvdcdc36/2005 | A/Equine/Pennsylvania/1/2007 |
| A/Chicken/Chile/176822/2002 | A/Chicken/Vietnam/Ncvdcdc37/2005 | A/Equine/Prague/1956 |
| A/Chicken/Chile/184240-4957/2002 | A/Chicken/Vietnam/Ncvdcdc42/2005 | A/Equine/Texas/2004 |
| A/Chicken/Egypt/A10543A/2015 | A/Chicken/Vietnam/Ncvdcdc52/2005 | A/Equine-2/Miami/1963 |
| A/Chicken/Egypt/A10758D/2015 | A/Chilean Teal/Chile/9/2013 | A/Goose/Bangladesh/25169/2015 |
| A/Chicken/Egypt/A1093D/2015 | A/Chukar/Shantou/22116/2005 | A/Goose/Egypt/M2794A/2011 |
| A/Chicken/Egypt/B9040A/2013 | A/Chukar/Shantou/2777/2015 | A/Goose/Hong Kong/631/2009 |
| A/Chicken/Egypt/D10553A/2015 | A/Common Buzzard/Bulgaria/38Wb/2010 | A/Gull/Delaware/428/2009 |
| A/Chicken/Egypt/D10561/2015 | A/Common Goldeneye/Iowa/3192/2009 | A/Gull/Maryland/704/1977 |
| A/Chicken/Egypt/D10565A/2015 | A/Common Magpie/Hong Kong/5052/2007 | A/Gyrfalcon/Washington/41088-6/2014 |
| A/Chicken/Egypt/D10705/2015 | A/Crested Myna/Hong Kong/8381/2012 | A/Hong Kong/1073/1999 |
| A/Chicken/Egypt/D10975/2015 | A/Denver/1/1957 | A/Hong Kong/156/1997 |
| A/Chicken/Egypt/D7663A/2013 | A/Duck/Alberta/35/1976 | A/Hong Kong/33982/2009 |
| A/Chicken/Egypt/F10533D/2015 | A/Duck/Alberta/60/1976 | A/Hong Kong/5942/2013 |
| A/Chicken/Egypt/F9514A/2014 | A/Duck/Bangladesh/1052/2007 | A/Hong Kong/69955/2008 |
| A/Chicken/Egypt/M7217B/2013 | A/Duck/Bangladesh/1283/2008 | A/Hong Kong/8/1968 |
| A/Chicken/Egypt/Q1089E/2010 | A/Duck/Bangladesh/1293/2008 | A/Indiana/14/2012 |
| A/Chicken/Egypt/Q10937B/2015 | A/Duck/Bangladesh/1559/2009 | A/Indiana/16/2012 |
| A/Chicken/Egypt/S10489C/2015 | A/Duck/Bangladesh/1575/2009 | A/Indiana/65/2012 |
| A/Chicken/Egypt/S10598E/2015 | A/Duck/Bangladesh/1746/2010 | A/Indiana/8/2011 |
| A/Chicken/Egypt/S3806B/2011 | A/Duck/Chabarovsk/1610/1972 | A/Japan/305/1957 |
| A/Chicken/Egypt/S4454B/2011 | A/Duck/Czech/1956 | A/Japanese White-Eye/Hong Kong/1038/2006 |
| A/Chicken/Egypt/S4456B/2011 | A/Duck/Egypt/C9787/2014 | A/Pintail/Alberta/293/1977 |
| A/Chicken/Egypt/S5018B/2012 | A/Duck/England/1956 | A/Quail/Bangladesh/19250/2013 |
| A/Chicken/Hong Kong/Cra45/2010 | A/Duck/Germany/1215/1973 | A/Quail/California/K1400794/2014 |
| A/Large-Billed Crow/Hong Kong/497/2011 | A/Mallard/Ohio/14Os2758/2014 | A/Quail/Egypt/D9842/2014 |
| A/Laughing Gull/ DE/12/2006 | A/Mallard/Ohio/14Os823/2015 | A/Quail/Hong Kong/G1/1997 |
| A/Laughing Gull/ DE/156/2004 | A/Mallard/Ohio/1688/2009 | A/Quail/Italy/1117/1965 |
| A/Laughing Gull/ DE/22/2002 | A/Mallard/PA/454069-9/2006 | A/Quail/Lebanon/272/2010 |
| A/Laughing Gull/ DE/45/2005 | A/Mallard/Ramon/79/14T | A/Quail/Lebanon/273/2010 |
| A/Laughing Gull/ Delaware/5/2003 | A/Mallard/Republic Of Georgia/4/2010 | A/Red Knot/Delaware Bay/240/1994 |
| A/Laughing Gull/Delaware Bay/94/1995 | A/Mallard/Wisconsin/4218/2009 | A/Red Knot/Delaware/239/2015 |
| A/Lesser Noddy/Western Australia/2371/1983 | A/Mallard/Wisconsin/4230/2009 | A/Red Knot/Delaware/259/1994 |
| A/Mallard/Alberta/114/1999 | A/Michigan/20/2012 | A/Red Knot/Delaware/269/2015 |
| A/Mallard/Alberta/125/1999 | A/Mississippi/3/2001 Mut H275Y | A/Red Knot/Delaware/541/1988 |
| A/Mallard/Alberta/162/2007 | A/Mississippi/3/2001 Wt 275H | A/Redhead/Alberta/192/2002 |
| A/Mallard/Alberta/167/2010 | A/Mule Duck/Bulgaria/237/2011 | A/Redheaded Duck/Minnesota/Sg-00123/2007 |
| A/Mallard/Alberta/174/2010 | A/Mule Duck/Bulgaria/328/2011 | A/Ring-Billed Gull/Quebec/02434-1/2009 |
| A/Mallard/Alberta/177/2004 | A/Netherlands/219/2003 | A/Ruddy Turnstone/Delaware Bay/39/1994 |
| A/Mallard/Alberta/203/1992 | A/New Caledonia/20/1999 | A/Ruddy Turnstone/Delaware/105/2007 |
| A/Mallard/Alberta/21/2014 | A/Northern Pintail/CA/44242-758/2006 | A/Ruddy Turnstone/Delaware/108/2007 |
| A/Mallard/Alberta/243/2006 | A/Northern Pintail/Missouri/319/2009 | A/Ruddy Turnstone/Delaware/115/2011 |
| A/Mallard/Alberta/27/2001 | A/Northern Pintail/Washington/40964/2014 | A/Ruddy Turnstone/Delaware/116/1998 |
| A/Mallard/Alberta/307/2012 | A/Northern Shoveler/Mississippi/09Os025/2009 | A/Ruddy Turnstone/Delaware/117/2011 |
| A/Mallard/Alberta/34/2001 | A/Northern pintail/WA/40564/2014 | A/Ruddy Turnstone/Delaware/124/2007 |
| A/Mallard/Alberta/341/2012 | A/Ohio/20/2012 | A/Ruddy Turnstone/Delaware/130/1999 |
| A/Mallard/Alberta/383/2009 | A/Ohio/36/2012 | A/Ruddy Turnstone/Delaware/134/1999 |
| A/Mallard/Alberta/435/2013 | A/Ohio/44/2012 | A/Ruddy Turnstone/Delaware/215/1991 |
| A/Mallard/Alberta/54/1993 | A/Ohio/47/2012 | A/Ruddy Turnstone/Delaware/237/1991 |
| A/Mallard/Alberta/579/2010 | A/Ohio/56/2012 | A/Ruddy Turnstone/Delaware/253/2011 |
| A/Mallard/Alberta/58/1989 | A/Ohio/83/2012 | A/Ruddy Turnstone/Delaware/262/2006 |
| A/Mallard/Alberta/747/2015 | A/Ostrich/Zimbabwe/222/1996 | A/Ruddy Turnstone/Delaware/268/2011 |
| A/Mallard/Arkansas/1C/2001 | A/Parakeet/Netherlands/267497/1994 | A/Ruddy Turnstone/Delaware/274/2009 |
| A/Mallard/Astrakhan/263/82 | A/Pennsylvania/17/2012 | A/Ruddy Turnstone/Delaware/282/2006 |
| A/Mallard/Illinois/10Os4334/2010 | A/Peregrine Falcon/Hong Kong/4955/2015 | A/Ruddy Turnstone/Delaware/282/2011 |
| A/Mallard/Ireland/Pv46B/1993 | A/Pheasant/New Jersey/30739-9/2000 | A/Ruddy Turnstone/Delaware/284/2006 |
| A/Mallard/Italy/80/1993 | A/Pheasant/Shantou/2785/2015 | A/Ruddy Turnstone/Delaware/431/2011 |
| A/Mallard/Korea/W452/2014 | A/Pheasant/United Arab Emirates/D1307.B/2011 | A/Ruddy Turnstone/Delaware/503/2011 |
| A/Mallard/Minnesota/346250/2000 | A/Pheasant/Washington/373/49/1985 | A/Ruddy Turnstone/New Jersey/A107-283/2007 |
| A/Mallard/Netherlands/12/2000 | A/Pigeon/Shantou/3577/2015 | A/Turkey/MN/1/1988 |
| A/Mallard/Ohio/14Os0821/2015 | A/Shoveler/Egypt/845/2004 | A/Turkey/North Dakota/11419-1/15 |
| A/Ruddy Turnstone/NJ/Ai11-1678/2011 | A/Snow Goose/Cc15-84B/2015 | A/Turkey/Ontario/6118/1968 |
| A/Ruddy Turnstone/Virginia/2297/1988 | A/Songbird/Hong Kong/Sb18/2001 | A/Turkey/Virginia/505477-18/2007 |
| A/Sanderling/Delaware/280/2015 | A/Swine/1976/1931 | A/Turkey/Wisconsin/1/1966 |
| A/Sanderling/Delaware/449/2006 | A/Swine/Minnesota/3908-2/2011 | A/Vietnam/1203/2004 |
| A/Santiago/5248/2008 | A/Swine/North Carolina/0666/2011 | A/Vietnam/Jp20-2/2005 |
| A/Seal/Massachusetts/1/1980 | A/Swine/North Carolina/0668/2011 | A/Virginia/01/2006 |
| A/Shanghai/1/2013 | A/Swine/North Carolina/32760/2007, | A/White-Faced Whistling Duck/Colombia/1/2011 |
| A/Shearwater/Australia/1/1973 | A/Swine/North Carolina/44897/2009, | A/Wisconsin/28/2012 |
| A/Shearwater/Australia/2576/1979 | A/Swine/North Carolina/52796/2006 | Rg-A/Swine/Nc/18162/2002 |
| A/Shorebird/Delaware Bay/211/1994 | A/Swine/North Carolina/88708/2000 | B/Brisbane/60/2008 |
| A/Shorebird/Delaware Bay/216/1999 | A/Swine/Ohio/09Sw1477/2009 | B/Cambodia/30/2011 |
| A/Shorebird/Delaware Bay/230/2009 | A/Swine/Ohio/09Sw1484E/2009 | B/Florida/02/2006 |
| A/Shorebird/Delaware Bay/31/1996 | A/Swine/Ohio/09Sw73E/2009 | B/Georgia/01/2011 |
| A/Shorebird/Delaware Bay/338/2009 | A/Swine/Ohio/09Sw79M/2009 | B/Malaysia/2506/2004 |
| A/Shorebird/Delaware Bay/63/1996 | A/Swine/Ohio/09Sw83E/2009 | B/New Jersey/01/2012 |
| A/Shorebird/Delaware/127/2003 | A/Swine/Ohio/11Sw87/2011 | B/North Carolina/03/2011 |
| A/Shorebird/Delaware/133/2002 | A/Swine/Tennessee/26/1977 | B/Nevada/03/2011 |
| A/Shorebird/Delaware/141/2002 | A/Swine/Texas/4199-2/1998 | B/Texas/02/2013 |
| A/Shorebird/Delaware/172/2006 | A/Shoveler/Egypt/597/2004 | B/Victoria/304/2006 |
| A/Shorebird/Delaware/192/1998 | A/Taiwan/T1.4/2013 | B/Bangladesh/3333/2007 |
| A/Shorebird/Delaware/218/2015 | A/Teal/Egypt/0457/2003 | A/Turkey/Minnesota/110915-1/2015 |
| A/Shorebird/Delaware/230/2000 | A/Teal/Egypt/431/2003 | A/Turkey/Minnesota/11668-1/2015 |
| A/Shorebird/Delaware/231/2003 | A/Teal/Egypt/677/2004 | B/Bangladesh/9673/2009 |
| A/Shorebird/Delaware/261/2003 | A/Teal/Egypt/835/2004 | B/Brisbane/03/2007 |
| A/Shorebird/Delaware/277/2000 | A/Texas/35/2008) | B/Chongqing/Yongchuan18/2007 |
| A/Shorebird/Delaware/28/1995 | A/Turkey/California/K1500529/2015 | B/Finland/39/2010 |
| A/Shorebird/Delaware/282/2011 | A/Turkey/Egypt/7/2007 | B/Florida/04/2006 |
| A/Shorebird/Delaware/318/2011 | A/Turkey/Egypt/S6405C/2012 | B/Florida/07/2004 |
| A/Shorebird/Delaware/464/2011 | A/Turkey/Indiana/1573-2/2016 | B/Fujian/Gulou/1553/2011 |
| A/Shorebird/Delaware/472/2007 | A/Turkey/Ireland/Pv74/1995 | B/Massachusetts/02/2012 |
| A/Shorebird/Delaware/53/2002 | A/Turkey/Italy/6423-1/1999 | B/Pennsylvania/07/2007 |
| A/Shorebird/Delaware/552/2006 | A/Turkey/Massachusetts/3740/1965 | B/Phuket/3073/2013 |
| A/Shorebird/Delaware/554/2007 | A/Turkey/Minnesota/037767/2009 | B/Texas/06/2011 |
| A/Shorebird/Delaware/75/2004 | A/Turkey/Minnesota/10777/2015 | B/Wisconsin/1/2010 |
| A/Shoveler/Egypt/0600/2004 |  |  |

| **Table S2:** composition of the tier 1 neural networks training set | | | | |
| --- | --- | --- | --- | --- |
| **Category** | **# images** | **# unique strains** | **# unique subtypes**  **(if applicable)** | **%** |
| A/H1N1 pdm 2009 | 446 | 48 | NA | 14.8% |
| A/Seasonal H3N2 | 369 | 26 | NA | 12.3% |
| A/Non-seasonal | 768 | 318 | 63 | 25.6% |
| B/Yamagata | 244 | 13 | NA | 8.1% |
| B/Victoria | 287 | 10 | NA | 9.6% |
| Clinical negatives | 472 | NA | NA | 15.7% |
| Other negative controls | 419 | NA | NA | 13.9% |
| **Total** | **3005** | **415** | **NA** | 100.0% |

| **Table S3: List of influenza A strains included in training tier 2 neural networks** | | | | | | | | | | |
| --- | --- | --- | --- | --- | --- | --- | --- | --- | --- | --- |
| A/American Green-Winged Teal/Mississippi/300/2010 | | | | | | A/Shorebird/Delaware Bay/127/2003 | | | | |
| A/Anhui/01/2005 | | | | | | A/Duck/Bangladesh/1746/2010 | | | | |
| A/Anhui/2/2005 | | | | | | A/Duck/Chabarovsk/1610/1972 | | | | |
| A/Aquatic Bird/Hong Kong/D125/2002 | | | | | | A/Duck/England/36254/2014 | | | | |
| A/Arizona/07/2007 | | | | | | A/Duck/Germany/1215/1973 | | | | |
| A/Avian/New York/11678-4/2005 | | | | | | A/Duck/Kulon Progo/Bb Vet/Ix/2004 | | | | |
| A/Blue-Winged Teal/Alberta/221/1978 | | | | | | A/Duck/Memphis/546/1974 | | | | |
| A/Blue-Winged Teal/Alberta/346/2007 | | | | | | A/Duck/Pennsylvania/10218/1984 | | | | |
| A/Blue-Winged Teal/Illinois/10Os1546/2010 | | | | | | A/Duck/Ukraine/1963 | | | | |
| A/Blue-Winged Teal/Illinois/10Os1563/2010 | | | | | | A/Environment/Vietnam/Ncvdcdc53/2005 | | | | |
| A/Blue-Winged Teal/Iowa/10Os2411/2010 | | | | | | A/Environment/Vietnam/Ncvdcdc54/2005 | | | | |
| A/Blue-Winged Teal/Iowa/10Os2411/2010 | | | | | | A/Equine/Kentucky/4/2011 | | | | |
| A/Blue-Wingedteal/Texas/G77/2007 | | | | | | A/Equine/New York/452/2003 | | | | |
| A/Brisbane/59/2007 | | | | | | A/Equine/Pennsylvania/1/2007 | | | | |
| A/Camel/Mongolia/335/2012 | | | | | | A/Equine/Prague/1956 | | | | |
| A/Canine/Florida/89911,2/2006 | | | | | | A/Equine/Texas/2004 | | | | |
| A/Canine/New York/100525,1/2006 | | | | | | A/Equine-2/Miami/1963 | | | | |
| A/Canine/New York/4732/2012 | | | | | | A/Fujian Gulou/1896/2009 | | | | |
| A/Canine/Virginia/93653/2009 | | | | | | A/Goose/Yunnan/5539/2005 | | | | |
| A/Chelyabinsk/01/2006 | | | | | | A/Hong Kong/1073/1999 | | | | |
| A/Chicken/Hong Kong/G9/1997 | | | | | | A/Hong Kong/5942/2013 | | | | |
| A/Chicken/Ny/Sg-00425/2004 | | | | | | A/Indiana/08/2011 | | | | |
| A/Chicken/Vietnam/Ncvd-016/2008 | | | | | | A/Indiana/14/2012 | | | | |
| A/Chicken/Vietnam/Ncvdcdc33/2005 | | | | | | A/Indiana/16/2012 | | | | |
| A/Chicken/Vietnam/Ncvdcdc36/2005 | | | | | | A/Indiana/65/2012 | | | | |
| A/Chicken/Vietnam/Ncvdcdc37/2005 | | | | | | A/Italy/3/2013 | | | | |
| A/Chicken/Vietnam/Ncvdcdc42/2005 | | | | | | A/Japan/305/1957 | | | | |
| A/Chicken/Vietnam/Ncvdcdc52/2005 | | | | | | A/Laughing Gull/Delaware Bay/94/1995 | | | | |
| A/Common Goldeneye/Iowa/3192/2009 | | | | | | A/Laughing Gull/Delaware/156/2004 | | | | |
| A/Duck/Alberta/35/1976 | | | | | | A/Mallard/Alberta/114/1999 | | | | |
| A/Duck/Bangladesh/1052/2007 | | | | | | A/Mallard/Alberta/125/1999 | | | | |
| A/Duck/Bangladesh/1283/2008 | | | | | | A/Mallard/Alberta/177/2004 | | | | |
| A/Duck/Bangladesh/1293/2008 | | | | | | A/Mallard/Alberta/203/1992 | | | | |
| A/Duck/Bangladesh/1559/2009 | | | | | | A/Mallard/Alberta/307/2012 | | | | |
| A/Duck/Bangladesh/1575/2009 | | | | | | A/Mallard/Alberta/435/2013 | | | | |
| A/Mallard/Alberta/58/1989 | | | | | | A/Mallard/Alberta/54/1993 | | | | |
| A/Mallard/Illinois/10Os4334/2010 | | | | | | A/Shorebird/Delaware Bay/127/2003 | | | | |
| A/Mallard/Netherlands/12/2000 | | | | | | A/Shorebird/Delaware Bay/211/1994 | | | | |
| A/Mallard/Ohio/1688/2009 | | | | | | A/Shorebird/Delaware Bay/31/1996 | | | | |
| A/Mallard/Pa/454069-9/2006 | | | | | | A/Shorebird/Delaware/172/2006 | | | | |
| A/Mallard/Republic of Georgia/4/2010 | | | | | | A/Shorebird/Delaware/192/1998 | | | | |
| A/Michigan/20/2012 | | | | | | A/Shorebird/Delaware/28/1995 | | | | |
| A/Minnesota/11/2010 | | | | | | A/Shoveler/Egypt/0600/2004 | | | | |
| A/Mississippi/3/2001 Mut H275Y | | | | | | A/Shoveler/Egypt/597/2004 | | | | |
| A/Mississippi/3/2001 Wt 275H | | | | | | A/Shoveler/Egypt/845/2004 | | | | |
| A/Mule Duck/Bulgaria/237/2011 | | | | | | A/Swine/1976/1931 | | | | |
| A/Mule Duck/Bulgaria/328/2011 | | | | | | A/Swine/Illinois/4L013/2016 | | | | |
| A/New Caledonia/20/1999 | | | | | | A/Swine/Illinois/4L036/2015 | | | | |
| A/Northern Pintail/Washington/40964/2014 | | | | | | A/Swine/Minnesota/3908-2/11 | | | | |
| A/Ohio/20/2012 | | | | | | A/Swine/North Carolina/32760/2007 | | | | |
| A/Ohio/36/2012 | | | | | | A/Swine/North Carolina/44897/2009 | | | | |
| A/Ohio/44/2012 | | | | | | A/Swine/North Carolina/52796/2006 | | | | |
| A/Ohio/47/2012 | | | | | | A/Swine/North Carolina/88708/2000 | | | | |
| A/Ohio/56/2012 | | | | | | A/Swine/Ohio/09Sw1477/2009 | | | | |
| A/Ohio/83/2012 | | | | | | A/Swine/Ohio/09Sw1484E/2009 | | | | |
| A/Pennsylvania/17/2012 | | | | | | A/Swine/Ohio/09Sw73E/2009 | | | | |
| A/Pheasant/United Arab Emirates/D1307.B/2011 | | | | | | A/Swine/Ohio/09Sw79M/2009 | | | | |
| A/Pheasant/Washington/373/49/1985 | | | | | | A/Swine/Ohio/09Sw83E/2009 | | | | |
| A/Pintail/Alberta/293/1977 | | | | | | A/Swine/Ohio/11Sw87/2011 | | | | |
| A/Quail/Lebanon/272/2010 | | | | | | A/Teal/Egypt/0457/2003 | | | | |
| A/Red Knot/Delaware Bay/240/1994 | | | | | | A/Teal/Egypt/431/2003 | | | | |
| A/Redhead/Alberta/192/2002 | | | | | | A/Teal/Egypt/677/2004 | | | | |
| A/Ring-Billed Gull/Quebec/02434-1/2009 | | | | | | A/Teal/Egypt/835/2004 | | | | |
| A/Ruddy Turnstone/Delaware Bay/39/1994 | | | | | | A/Texas/35/2008 | | | | |
| A/Ruddy Turnstone/Delaware/130/1999 | | | | | | A/Turkey/Virginia/505477-1820/2007 | | | | |
| A/Ruddy Turnstone/Delaware/134/1999 | | | | | | A/Turkey/Wisconsin/1/1966 | | | | |
| A/Ruddy Turnstone/Delaware/274/2009 | | | | | | A/Vietnam/Jp20-2/2005 | | | | |
| A/Ruddy Turnstone/New Jersey/Ai11-1678/2011 | | | | | | A/Virginia/01/2006 | | | | |
| A/Ruddy Turnstone/Virginia/2297/1988 | | | | | | A/White-Faced Whistling Duck/Colombia/1/2011 | | | | |
| A/Santiago/5248/2008 | | | | | | A/Wisconsin/28/2012 | | | | |
|  | | | | | | | | |  |  |
| **Table S4:** Subtype Composition of Tier 2 (HA/NA Subtyping) Neural Network Training Set | | | | | | | | | | |
| **HA subtype** | | **# images** | **# unique strains** | **%** | **NA subtype** | **# images** | | **# unique strains** | | **%** |
| H1 | | 353 | 23 | 23.9 | N1 | 447 | | 35 | | 30.2 |
| H3 | | 290 | 46 | 19.6 | N2 | 441 | | 44 | | 31.9 |
| H5 | | 279 | 24 | 18.9 | N7 | 115 | | 8 | | 7.8 |
| H7 | | 237 | 13 | 16.0 | N8 | 212 | | 24 | | 14.3 |
| H9 | | 209 | 10 | 14.1 | N9 | 169 | | 12 | | 11.4 |
| Hx (all other) | | 111 | 24 | 7.5 | Nx (all other) | 65 | | 63 | | 4.4 |
| **Total** | | **1479** | **140*** | **100** | **Total** | **1479** | | **140*** | | **100** |
| *Strain information was not available for 11 viruses included, and therefore are characterized only by subtype and are not included in the number of unique strains. | | | | | | | | | | |

|  | |
| --- | --- |
| **Table S5: List of influenza A strains included the naïve test set** | |
| A/American Green-Winged Teal/Mississippi/300/2010 | A/Mallard/Italy/80/1993 |
| A/Black Headed Gull/Hong Kong/84/2012 | A/Mallard/Netherlands/12/2000 |
| A/Black-Legged Kittiwake/Quebec/02838-1/2009 | A/Mallard/Ohio/14Os2758/2014 |
| A/Blue-Winged Teal/Alberta/346/2007 | A/Mallard/Ohio/1688/2009 |
| A/Blue-Winged Teal/Illinois/10Os1546/2010 | A/Mallard/Ramon/79/14T |
| A/Cambodia/X0810301/2013 | A/Mallard/Republic of Georgia/4/2010 |
| A/Canvasback/Alberta/276/2005 | A/Mallard/Wisconsin/4218/2009 |
| A/Chick/Germany/N/1949 | A/Mallard/Wisconsin/4230/2009 |
| A/Chicken/Bangladesh/23974/2014 | A/Michigan/20/2012 |
| A/Chicken/Beijing/1/1994 | A/Netherlands/33/2003 |
| A/Chicken/Chile/176822/2002 | A/Northern Pintail/Missouri/319/2009 |
| A/Chicken/Egypt/A10758D/2015 | A/Northern Shoveler/ Mississippi/09Os025/2009 |
| A/Chicken/Egypt/B9040A/2013 | A/Ohio/20/2012 |
| A/Chicken/Egypt/F9514A/2014 | A/Ohio/36/2012 |
| A/Chicken/Egypt/M7217B/2013 | A/Ohio/44/2012 |
| A/Chicken/Egypt/Q1089E/2010 | A/Ostrich/Zimbabwe/222/1996 |
| A/Chicken/Egypt/Q10937B/2015 | A/Parakeet/Netherlands/267497/1994 |
| A/Chicken/Egypt/S3806B/2011 | A/Peregrine Falcon/Hong Kong/4955/2015 |
| A/Chicken/Hong Kong/Nt10/2011 | A/Pheasant/Nj/30739-9/2000 |
| A/Chicken/Hunan/2246/2006 | A/Quail/Ca/K1400794/2014 |
| A/Chicken/Italy/1285/2000 | A/Quail/Hong Kong/G1/1997 |
| A/Chicken/Italy/312/1997 | A/Quail/Italy/1117/1965 |
| A/Chicken/Italy/9097/1997 | A/Quail/Lebanon/272/2010 |
| A/Chicken/Jalisco/12283/2012 | A/Red Knot/De/259/1994 |
| A/Chicken/New York/116124/2003 | A/Red Knot/Delaware/541/1988 |
| A/Chicken/Ny/116124/2003 | A/Redhead/Alberta/192/2002 |
| A/Chicken/Qalubia-Egypt/1/2006 | A/Redheaded Duck/Minnesota/Sg-00123/2007 |
| A/Chicken/Qalubia-Egypt/1/2008 | A/Ring-Billed Gull/Quebec/02434-1/2009 |
| A/Chicken/Vietnam/Ncvd-14-A324/2014 | A/Ruddy Turnstone/De/105/2007 |
| A/Chicken/Vietnam/Ncvdcdc23/2005 | A/Ruddy Turnstone/De/117/2011 |
| A/Chicken/Vietnam/Ncvdcdc3/2005 | A/Ruddy Turnstone/Delaware Bay/39/1994 |
| A/Chicken/Vietnam/Ncvdcdc49/2005 | A/Ruddy Turnstone/Delaware/215/1991 |
| A/Chukar/Shantou/22116/2005 | A/Ruddy Turnstone/Delaware/237/1991 |
| A/Common Buzzard/Bulgaria/38Wb/2010 | A/Ruddy Turnstone/Delaware/282/2011 |
| A/Common Goldeneye/Iowa/3192/2009 | A/Ruddy Turnstone/Delaware/290/2006 |
| A/Common Magpie/Hong Kong/5052/2007 | A/Ruddy Turnstone/Delaware/293/2006 |
| A/Common Magpie/Hong Kong/645/2006 | A/Ruddy Turnstone/Delaware/431/2011 |
| A/Crested Myna/Hong Kong/8381/2012 | A/Ruddy Turnstone/Delaware/510/1988 |
| A/Duck/Alberta/35/1976 | A/Ruddy Turnstone/Virginia/2297/1988 |
| A/Duck/Alberta/60/1976 | A/Seal/Massachusetts/1/1980 |
| A/Duck/Bangladesh/19097/2013 | A/Shanghai/1/2013 |
| A/Duck/Chabarovsk/1610/1972 | A/Shearwater/Australia/1/1973 |
| A/Duck/Czech/1956 | A/Shorebird/Delaware Bay/211/1994 |
| A/Duck/England/1956 | A/Shorebird/Delaware Bay/216/1999 |
| A/Duck/Germany/1215/1973 | A/Shorebird/Delaware Bay/230/2009 |
| A/Duck/Hokkaido/69/2000 | A/Shorebird/Delaware Bay/31/1996 |
| A/Duck/Hong Kong/Y280/1997 | A/Shorebird/Delaware Bay/338/2009 |
| A/Duck/Minnesota/1525/1981 | A/Shorebird/Delaware Bay/63/1996 |
| A/Duck/Pennsylvania/10218/1984 | A/Shorebird/Delaware/101/2004 |
| A/Duck/Potsdam/S28716/1988 | A/Shorebird/Delaware/124/2001 |
| A/Duck/Ukraine/1963 | A/Shorebird/Delaware/133/2002 |
| A/Egret/Egypt/1162-Namru3/2006 | A/Shorebird/Delaware/230/2000 |
| A/Egypt/N04915/2014 | A/Shorebird/Delaware/246/2003 |
| A/Equine/California/191/2003 | A/Shorebird/Delaware/260/2000 |
| A/Equine/Massachusetts/213/2003 | A/Shorebird/Delaware/277/2000 |
| A/Equine/Pennsylvania/1/2007 | A/Shorebird/Delaware/282/2011 |
| A/Equine-2/Miami/1963 | A/Shorebird/Delaware/309/2008 |
| A/Florida/03/2006 | A/Shorebird/Delaware/318/2011 |
| A/Goose/Bangladesh/25169/2015 | A/Shorebird/Delaware/472/2007 |
| A/Goose/Egypt/M2794A/2011 | A/Shorebird/Delaware/6/2002 |
| A/Gray Plover/Chile/C1313/2015 | A/Shorebird/Delaware/75/2004 |
| A/Gull/Delaware/428/2009 | A/Snow Goose/Cc15-84B/2015 |
| A/Gull/Maryland/704/1977 | A/Solomon Islands/3/2006 |
| A/Hong Kong/156/1997 | A/Songbird/Hong Kong/Sb18/2001 |
| A/Hong Kong/308/2014 | A/South Dakota/06/2007 |
| A/Hong Kong/33982/2009 | A/St. Petersburg/8/2006 |
| A/Indiana/10/2011 | A/Swine/North Carolina/0666/2011 |
| A/Indonesia/Nihrd11771/2011 | A/Swine/North Carolina/0668/2011 |
| A/Japan/305/1957 | A/Swine/North Carolina/32760/2007 |
| A/Japanese White-Eye/Hong Kong/1038/2006 | A/Swine/North Carolina/44897/2009 |
| A/Large-Billed Crow/Hong Kong/497/2011 | A/Swine/North Carolina/52796/2006 |
| A/Laughing Gull/Delaware/12/2006 | A/Swine/North Carolina/88708/2000 |
| A/Laughing Gull/Delaware/2/2002 | A/Swine/Ohio/09Sw1477/2009 |
| A/Laughing Gull/Delaware/42/2006 | A/Swine/Ohio/09Sw1484E/2009 |
| A/Laughing Gull/Delaware/45/2005 | A/Swine/Tennessee/26/1977 |
| A/Lesser Noddy/Western Australia/2371/1983 | A/Swine/Texas/4199-2/1998 |
| A/Mallard Duck/Alberta/743/1983 | A/Swine/Wisconsin/125/1997 |
| A/Mallard/Alberta/125/1999 | A/Teal/Egypt/665/2004 |
| A/Mallard/Alberta/167/2010 | A/Turkey/Egypt/7/2007 |
| A/Mallard/Alberta/194/1992 | A/Turkey/Egypt/S6405C/2012 |
| A/Mallard/Alberta/203/1992 | A/Turkey/Italy/6423-1/1999 |
| A/Mallard/Alberta/243/2006 | A/Turkey/Massachusetts/3740/1965 |
| A/Mallard/Alberta/26/2001 | A/Turkey/Minnesota/037767/2009 |
| A/Mallard/Alberta/27/2001 | A/Turkey/Minnesota/1/1988 |
| A/Mallard/Alberta/34/2001 | A/Turkey/Ontario/6118/1968 |
| A/Mallard/Alberta/35/2001 | A/Vietnam/1194/2004 |
| A/Mallard/Alberta/383/2009 | A/Vietnam/1203/2004 |
| A/Mallard/Arkansas1C/2001 | A/Wedge-Tailed Shearwater/Western Australia/2327/1983 |
| A/Mallard/Astrakhan/263/1982 | A/Wedge-Tailed Shearwater/Western Australia/2576/1979 |
| A/Mallard/Illinois/10Os4334/2010 | RG-A/swine/NC/18162/2002 |
